# Cellular Stress Responses and Gut Microbiota in Inflammatory Bowel Disease

**DOI:** 10.1155/2018/7192646

**Published:** 2018-06-20

**Authors:** Siyan “Stewart” Cao

**Affiliations:** Department of Medicine, University of California, San Francisco, San Francisco, CA 94143, USA

## Abstract

Progresses in the past two decades have greatly expanded our understanding of inflammatory bowel disease (IBD), an incurable disease with multifaceted and challenging clinical manifestations. The pathogenesis of IBD involves multiple processes on the cellular level, which include the stress response signaling such as endoplasmic reticulum (ER) stress, oxidative stress, and hypoxia. Under physiological conditions, the stress responses play key roles in cell survival, mucosal barrier integrity, and immunomodulation. However, they can also cause energy depletion, trigger cell death and tissue injury, promote inflammatory response, and drive the progression of clinical disease. In recent years, gut microflora has emerged as an essential pathogenic factor and therapeutic target for IBD. Altered compositional and metabolic profiles of gut microbiota, termed dysbiosis, are associated with IBD. Recent studies, although limited, have shed light on how ER stress, oxidative stress, and hypoxic stress interact with gut microorganisms, a potential source of stress in the microenvironment of gastrointestinal tract. Our knowledge of cellular stress responses in intestinal homeostasis as well as their cross-talks with gut microbiome will further our understanding of the pathogenesis of inflammatory bowel disease and probably open avenues for new therapies.

## 1. Introduction

Inflammatory bowel disease (IBD) affects more than 3.1 million people in the United States and 2.5 million in Europe and is increasing in incidence worldwide especially in regions such as Eastern and South Asia [1, 2]. IBD including Crohn's disease (CD) and ulcerative colitis (UC) are characterized by chronic intestinal inflammation as well as extraintestinal manifestations, which are likely triggered by a combination of genetic predispositions and environmental factors such as diet, infection, and medication use [3]. The complex pathophysiology of IBD involves multiple cell populations in the gastrointestinal tract and numerous signaling pathways from energy homeostasis to innate immune response. Cellular stress signaling, including endoplasmic reticulum (ER) stress, oxidative stress, and hypoxic stress, has been implicated in multiple human pathologies such as metabolic disease, neurodegenerative disease, cardiovascular disease, cancer, and autoimmune disease. Recent evidence suggests that stress signaling plays a key role in mucosal homeostasis in the gut. As an integral component of the gastrointestinal environment, the gut microbiome has been shown to promote intestinal health as a physiological stressor. Meanwhile, through complex interactions with the stress pathways in the host cells, these microorganisms may contribute to the pathogenesis of IBD by inducing cell death and differentiation, epithelial barrier breakdown, and inflammatory response. Recently, targeting cellular stress signaling and the gut microbiota have been proposed as new therapies for UC and CD. The understanding of both sides and the bridge between the two will likely spur the progresses in this arena.

## 2. Cellular Stress Signaling in Gastrointestinal Tract

### 2.1. ER Stress

Approximately one-third of all proteins synthesized in the cell travel across the ER where they are folded into their proper three-dimensional structures with posttranslational modifications including disulfide bond formation, glycosylation, hydroxylation, and lipidation. Protein folding in the ER is subject to numerous insults such as increased mRNA translation, elevated demand of protein secretion, genetic mutations of client proteins, deficiency of ER chaperones or foldases, ER redox or calcium perturbations, bacterial and viral infection, ATP depletion, nutrient deficiency, and pharmacological and toxicological insults. The accumulation of unfolded or misfolded proteins in the ER lumen activates the unfolded protein response (UPR), which is signaled through three ER transmembrane proteins: inositol-requiring enzyme 1*α* (IRE1*α*), dsRNA-activated protein kinase-related ER protein kinase (PERK), and activating transcription factor 6*α* (ATF6*α*). All three transmembrane sensors are maintained in an inactive state through interaction between their ER luminal domains and the protein chaperone immunoglobulin heavy chain-binding protein (BiP). A recent study showed that the ER luminal cochaperone ERdj4/DNAJB9 is required for the complex formation between BiP and the luminal domain of IRE1a, thereby maintaining IRE1a in a monomeric, inactive state in the absence of ER stress [4]. Upon ER stress, accumulated unfolded/misfolded proteins in the ER lumen bind and sequester BiP, thereby triggering dissociation of BiP from IRE1*α*, PERK, and ATF6*α* [5–7]. All three branches of the UPR have been involved in intestinal mucosal homeostasis and the pathogenesis of IBD.

The most evolutionarily conserved ER stress transducer is IRE1*α*, which contains both endoribonuclease and kinase domains within its cytosolic region. During ER stress, IRE1*α* activates via dimerization and auto-transphosphorylation, which then leads to removal of a 26-nucleotide intron from the mRNA encoding an active and stable transcription factor XBP1s. The activity of XBP1s has been linked to cellular proteostasis by inducing the expression of various genes involved in ER protein translocation, protein folding and modification, quality-control mechanisms, biogenesis of the ER and Golgi compartments, and ER-associated protein degradation (ERAD) [5, 6]. In addition to the cleavage of *Xbp1* mRNA, IRE1*α* regulates the UPR, cell death and differentiation, and inflammation via regulated IRE1a-dependent decay (RIDD) of various mRNAs and miRNAs, which was recently shown to orchestrate the survival of conventional dendritic cell in the gut [8]. Furthermore, IRE1*α* also cross-talks with a number of other stress-related signaling pathways including autophagy, nuclear factor-*κ*B (NF-*κ*B), mitogen-activated protein (MAP) kinases, and the c-Jun N-terminal kinase (JNK) pathways [6].

The IRE1*α*-XBP1 pathway has been well known to play an essential role in immunity and inflammation. XBP1 is required for the differentiation of plasma cells, dendritic cells, CD8^+^ T cells, and eosinophils, as well as the production of several cytokines in macrophages [9, 10]. XBP1 is one of the first UPR effectors to associate with IBD. Findings from both animal and human studies suggested an essential role of XBP1-mediated adaptive UPR signaling in the intestinal homeostasis. Loss of *Xbp1* in murine intestinal epithelial cells (IECs) hyperactivates IRE1*α*, which results in a NF-*κ*B and JNK-dependent inflammatory response. A recent study showed that the selective autophagy receptor optineurin may interact with and target IRE1*α* for degradation, thereby containing IRE1a aggregation and hyperactivation in IECs [11]. XBP1 deficiency leads to Paneth cell death, spontaneous small bowel inflammation, and susceptibility to bacterial infection [12]. Interestingly, ablation of *Xbp1* in Paneth cells alone (using *Defa6*-Cre) is sufficient to produce similar phenotype of spontaneous ileitis as observed in the previous model [13]. In addition to Paneth cells, the goblet cells in the large bowel were also affected by *Xbp1* deletion as shown by impaired mucin secretion. These animals were more susceptible to dextran sodium sulfate- (DSS-) induced colitis, which is probably caused by multiple mechanisms including the absence of XBP1-mediated adaptive UPR, proapoptotic effectors such as CCAAT-enhancer-binding protein homologous protein (CHOP), cellular inflammatory response in the settings of IRE1*α* activation, and its downstream signaling [14]. In colonic mucosal tissues from mice and humans, *Xbp1* was targeted by a colitis-induced miRNA, miR665, which was shown to promote apoptosis and DSS-induced colitis in mice [15]. A recent study suggested that loss of *Xbp1* in the IECs leads to increased expression of natural killer group 2 member D ligand (NKG2DL) mouse UL16-binding protein-like transcript 1 via CHOP, which likely contributes to the recruitment of NKG2D-expressing group 1 innate lymphoid cells and mucosal inflammation [16]. In addition, a human deep-sequencing study identified hypomorphic, rare variants of *XBP1* that associate with both CD and UC [12]. Despite that hyperactivation of IRE1*α* seems to contribute to mucosal inflammation, the deletion of *Ire1α* in murine IECs resulted in the loss of colonic goblet cells, impaired barrier function, and subsequent spontaneous colitis and rectal bleeding. There was a decreased *Xbp1* mRNA splicing, as expected, and an increased CHOP in the colonocytes of the mutant animals [14]. Although the IRE1*α* and XBP1 deficiencies in IECs cause mucosal inflammation from different mechanisms, it seems that both models involve proapoptotic UPR signaling such as CHOP. It would be interesting to test whether the ablation of *Chop* in IECs rescues the phenotypes (see more discussions below). It is also worthwhile to notice that *Xbp1* deletion produced more dramatic abnormalities in the small intestine, while the loss of IRE1*α* generated phenotype mainly in the large bowel. It is unclear whether this is due to the differential roles of IRE1*α* versus XBP1 in different IECs (Paneth cells versus goblet cells) or it is related to the microflora in specific animal facilities [17–19].

PERK is an ER transmembrane protein with a serine/threonine kinase domain on its cytosolic side. The activated PERK directly phosphorylates the eukaryotic mRNA translation initiation factor eIF2*α*, which dampens global protein synthesis and therefore relieving protein folding overload in the ER [5, 20, 21]. Meanwhile, the phosphorylated eIF2*α* undergoes selective translation of some mRNAs including that of activating transcription factor 4 (ATF4), which induces various genes involved in ER protein translocation and folding, antioxidative response, autophagy, and amino acid metabolism. ATF4 also engages apoptotic and inflammatory pathways through the transactivation of *Chop* and *Bcl-2* family members, as well as *Mcp1*, *Il6*, and *Tnfα* [5, 22]. The role of eIF2*α* phosphorylation in murine IECs was studied using mice with IEC-specific expression of nonphosphorylatable eIF2*α* [23]. Electron microscopy of Paneth cells in the mutant mice revealed a decreased number of secretory granules, a fragmented ER, and damaged mitochondria, which resembled the morphology of the Paneth cells deficient of autophagy mediator ATG16L1 or ATG5 [24]. The mutant mice exhibited a diminished secretion of lysozyme and cryptdins in their small bowel, which were likely responsible for the increased susceptibility to Salmonella infection. Interestingly, the ER-associated translation of *Lysozyme* and *Cryptdin* mRNAs was specifically impaired, which is associated with reduced expression of ER protein translocation machinery in the absence of eIF2a phosphorylation [5, 23]. A recent study showed that lysozyme is transported via secretory autophagy in Paneth cells, a process requiring bacteria-induced PERK-eIF2*α* activation [25]. In addition, eIF2*α* phosphorylation is linked to mucosal homeostasis in the large bowel. The unaffected mucosal tissue of UC patients exhibited decreased eIF2*α* phosphorylation, which may indicate a defective integrated stress response in colonic epithelial cells [26]. Most data so far support a protective role of eIF2*α* phosphorylation in IECs for intestinal homeostasis. In contrast, the overexpression of transcription factor *Chop* exacerbated DSS-induced colitis, which seems due to impaired epithelial cell proliferation rather than increased apoptosis [27]. Consistently, a recent study showed that the absence of CHOP alleviates bile duct ligation-induced loss of stemness in intestinal stem cells, although the underlying mechanism is unclear [28]. Besides ER stress, eIF2*α* phosphorylation serves as the regulatory hub of the so-called integrated stress response, which can also be initiated by three other serine/threonine eIF2*α* kinases in response to distinct stimuli including viral infection (via the double-stranded RNA-activated protein kinase, a.k.a. PKR), amino acid deprivation (via the general control nonderepressible 2, a.k.a. GCN2), and heme deficiency (via the heme-regulated eIF2*α* kinase, a.k.a. HRI) [6, 29]. PKR has been implicated in a broad spectrum of infectious and inflammatory processes in addition to its well-known antiviral response [30–33]. PKR is activated in murine colonic epithelial cells during DSS-induced colitis. The deletion of *Pkr* exacerbated epithelial cell death and colonic inflammation, which are likely due to reduced ER chaperones and other prosurvival pathways such as Akt/PKB and the signal transducer and activator of transcription 3 [34, 35]. A recent study linked GCN2 to intestinal inflammation. The genetic ablation of *Gcn2* in either IECs or CD11c^+^ antigen-presenting cells leads to Th17 cell activation and intestinal inflammation, which are likely due to impaired autophagy, elevated reactive oxygen species, and subsequent inflammasome activation [36].

Unlike IRE1*α* and PERK, ATF6*α* contains a basic leucine zipper transcription factor domain within its cytosolic region. Upon dissociation of BiP during ER stress, ATF6*α* translocates to the Golgi apparatus, where it is cleaved by endopeptidases S1P and S2P. The released ATF6*α* p50 fragment then travels to the nucleus and induces genes that are involved in protein folding and ERAD [7]. The *Atf6a*^−/−^ mice reconstituted with wild-type bone marrow cells are more vulnerable to DSS-induced colitis, likely due to diminished expression of ER chaperones including BiP and P58^IPK^ and increased proapoptotic UPR signaling including CHOP in the colonocytes [37].

### 2.2. Oxidative Stress

Oxygen metabolism produces reactive oxygen species (ROS) including superoxide, hydroxyl radicals, hydrogen peroxide, hypochlorous acid, and lipid hydroperoxides [38]. The electron transport chain in mitochondria generates a large proportion of ROS [39, 40]. Other sources of endogenous ROS in mammals include the ER, peroxisomes, nucleus as well as the cytosol, and extracellular matrix. Under physiological conditions, the cell is protected from ROS by its antioxidant capacity. However, an excessive ROS production or insufficient antioxidative response can cause lipid and protein modifications, DNA damage, altered membrane permeability, inflammatory response, and cell death, all of which can contribute to the pathogenesis of IBD [41–44]

Multiple cell types in the gastrointestinal tract can be both perpetrators and victims of oxidative stress. Induced by inflammatory cytokines, the IECs produce superoxide and nitric oxide that can damage cytoskeleton proteins and alter tight junctions, subsequently leading to barrier disruption and exacerbation of mucosal inflammation [45, 46]. Upon activation, neutrophils and macrophages in the intestine generate more ROS, which forms a vicious circle of mucosal injury and inflammation [47, 48]. Furthermore, oxidative stress can be initiated by environmental factors, such as luminal antigens, cigarette smoking, alcohol, drugs, xenobiotics, radiation, and chemotherapy, all of which contribute to IBD [3, 49]. Tobacco smoking is one of best known environmental risk factors for CD, whereas it is considered as an alleviating factor of UC. How smoking affects CD risks is not fully understood. Several mechanisms have been proposed. First of all, the gas and tar of cigarette smoke contain a large amount of ROS. Second, cigarette smoke can affect the ROS production in the cell. For example, metal ions from tobacco smoke promote the generation of highly reactive hydroxyl radical from hydrogen peroxide [50]. The activity of inducible nitric oxide synthase is elevated in the small intestine after nicotine administration, although the underlying mechanism remains unclear. Third, tobacco smoke can impair the antioxidative response via multiple mechanisms including the inactivation of superoxide dismutase [51–53]. In recent years, the genetic variations of several antioxidant enzyme genes have been associated with IBD. Some examples include the genes encoding superoxide dismutase 2, glutathione S-transferase, NAD(P)H:quinone oxidoreductase 1, paraoxonase 1, and nuclear factor- (erythroid-derived 2) like 2 [54–61]. Interestingly, the loss of peroxiredoxin 6, an important antioxidative protein, partially protected mice against both acute and chronic DSS-induced colitis, likely by promoting a compensatory elevation of other antioxidative enzymes in the gastrointestinal tract [62]. However, it is unclear whether peroxiredoxin 6 has similar function in different cell populations (e.g. IECs versus immune cells) during intestinal inflammation. This data suggests that the network of antioxidative mechanisms in the gut may be more complex than we previously appreciated.

### 2.3. Hypoxia

The association between mucosal hypoxia and intestinal inflammation has been demonstrated by hypoxic staining of mucosal tissues in animal models of colitis as well as elevated hypoxia-induced transcription factors in inflamed intestinal samples from individuals with IBD [63–68]. There is also an interesting finding that travelling to altitude > 2000 meters or taking flights increased the risk of IBD exacerbations within 4 weeks of travel, suggesting the role of hypoxia in the course of IBD [69]. Under normal conditions, there is a steep oxygen gradient from the intestinal lumen towards the submucosa in mammalian gastrointestinal tract [68, 70]. Intestinal mucosa becomes more hypoxic during inflammation due to increased oxygen consumption by infiltrated immune cells as well as decreased oxygen supply due to local microvascular occlusion and thrombosis [71, 72]. Mammalian cells have developed evolutionarily conserved mechanisms, including the prolyl hydroxylase domain protein- (PHD-) hypoxia-inducible factors (HIFs) pathway and NF-*κ*B, to code with hypoxic stress [73].

The degree of hypoxia in the GI tract was measured with different measures. The level of intestinal hypoxia under normal conditions, termed physiologic hypoxia, is indispensable for intestinal homeostasis [74]. Low-grade hypoxia promotes intestinal barrier function by inducing the expression of genes such as trefoil factor 3, mucin-3, multidrug resistance protein 1, CD39, CD73, and creatine kinases in IECs [75–78]. In addition to signal transduction via HIFs, PHD3 has been shown to stabilize the tight junction protein occludin in IECs by preventing its interaction with the E3 ligase Itch in a hydroxylase-independent manner [79]. Using a zebrafish model, intestinal *hif1ab* was shown to bind to a hypoxia-inducible response element of mammalian anterior gradient 2 (AGR2) that encodes an ER protein disulfide-isomerase [80]. *AGR2* is associated with IBD and required for the differentiation and maturation of intestinal goblet cells in mice [81–84]. Furthermore, physiological level hypoxia in IECs is important for the constitutive expression of *β* defensin, an antimicrobial peptide, and netrin-1 that impedes neutrophil infiltration into intestinal mucosa [85, 86]. In contrast, severe hypoxia can elicit an inflammatory response in the gut by targeting multiple cell types. IECs produce higher level of inflammatory cytokines such as TNF*α* and even enter apoptosis when the local oxygen drops below the physiological level [87, 88]. Mucosal immune cells are also affected by hypoxia during inflammation. Similar to IECs, pathologic hypoxia induces expression of proinflammatory cytokines in macrophages and dendritic cells [89–91]. Importantly, hypoxic macrophages were shown to downregulate autophagy in IECs, a protective signaling against mucosal inflammation. Upon hypoxia, HIF-1 induces Wnt1 expression in macrophages, which suppressed autophagy in cocultured epithelial cells via the activation of *β*-catenin and mTOR [92]. Knockout of the gene encoding PHD1, an inhibitor of the HIF signaling, drives macrophages towards an anti-inflammatory M2 phenotype and attenuates IL-1b production in dendritic cells in response to lipopolysaccharide [93]. In addition, dendritic cell-specific deletion of *Hif1α* in mice worsened DSS-induced colitis. This is likely due to impaired activation of Foxp3+ regulatory T cells in the settings of decreased thymic stromal lymphopoietin receptor and IL-10 in *Hif1a*^−/−^ dendritic cells [94]. Interestingly, hypoxia-challenged neutrophils may actually survive longer, thereby delaying the resolution of inflammation [95, 96]. Hypoxia also elicits the expression of *β*2 integrin that promotes leukocyte adhesion and extravasation [97]. Upon severe hypoxia, endothelial cells release proinflammatory cytokines, prostaglandins, platelet-activating factor, and P-selectin, all of which contribute to neutrophil recruitment and mucosal inflammation [98, 99].

## 3. Cellular Stress Responses Interact with Microbiome in Gastrointestinal Tract

A role of the gut microbiota in the pathogenesis of IBD has long been studied. In particular, both UC and DC are associated with dysbiosis, defined as a decrease in gut bacterial diversity due to a shift from commensal to potentially pathogenic species [100–102]. For example, the Firmicutes phylum usually decreases in the fecal samples of patients with CD compared to healthy individuals, while the Proteobacteria phylum including *Escherichia coli* is often increased in proportional abundance in patients with UC or CD [101, 103–109]. Studies of pediatric cohorts have revealed substantial differences in gut bacterial populations between diseased and healthy children [110–112]. Importantly, dysbiosis has also been observed in the stool and mucosal samples from newly diagnosed, treatment-naive children with CD [113], suggesting that dysbiotic alternations might precede the onset of clinical disease. The gut microbiota also includes viruses and fungi, whose roles in IBD have been increasingly appreciated. Bacteriophages are the predominant species of the gut virome as shown by metagenomic analyses of viral particles from human stool samples [114, 115]. Altered bacteriophage composition has been associated with IBD. In particular, expansion of *Caudovirales* bacteriophages was observed in patients with CD [116, 117]. A recent study suggests that some phages have immunomodulatory functions on human peripheral mononuclear cells by downregulating CD14, TLR4 while upregulating IL-10, IL-1R antagonist, and suppressor of cytokine signaling 3 [118]. However, whether bacteriophages in the gut have similar properties is unclear. There are very limited data about the role of eukaryotic viruses in IBD [119]. Similarly, changes in fungal composition in the gut have been reported in both fecal and mucosa samples. Animal studies suggest that fungi may affect intestinal homeostasis by regulating host metabolism, mucosal immune response, and other microorganisms in the gut [120], although a causal relationship between specific fungal species and IBD remains undetermined.

### 3.1. ER Stress and Gut Microbiota

**Figure 1:**
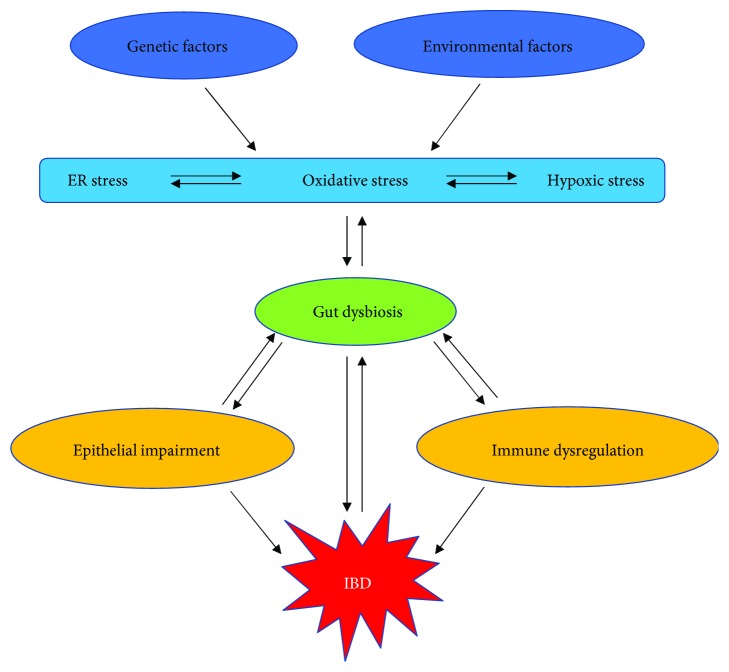
Cellular stress signaling interacts with the gut microbiome in the pathogenesis of IBD.

The interactions between bacteria and the ER in mammalian cells have long been recognized and investigated (Figure 1). As a biosynthetic factory of the cell, the ER is a nutrient-rich environment presumably free of antimicrobial or hydrolytic enzymes, making it a safe haven for the survival and proliferation of many intracellular bacteria. There are few studies so far that directly examined how gut commensals interact with the ER. However, several gastrointestinal pathogens have been shown to induce ER stress and the UPR both *in vitro* and *in vivo*. For example, *Helicobacter pylori* can elicit ER stress in gastric epithelial cells with its vacuolating cytotoxin VacA, which activates the PERK-eIF2*α*-CHOP pathway and causes mitochondrial damage and subsequent apoptosis [120]. ER stress induction is not restricted to ER-dwelling microorganisms. Secreted toxins enable extracellular bacteria to disrupt ER homeostasis without entering the cell. *Listeria monocytogenes* can activate all three branches of the UPR and downstream apoptotic signaling via secretion of cytolysin listeriolysin O (LLO), likely by disturbing ER calcium homeostasis through its pore-forming activity [121, 122]. Other bacteria produce and deliver proteins with enzymatic activities specifically targeting the UPR pathway. Shiga-toxigenic *Escherichia coli* secretes subtilase cytotoxin that cleaves the ER chaperone BiP, triggering the UPR activation and subsequent cell cycle arrest via translation attenuation and degradation of cyclin D1 [123–125]. Several gastrointestinal pathogens are known to exploit ER stress and the UPR for its survival and/or replication. ER stress may play a key role in the mucosal inflammatory response to microorganisms and their secreted molecules. For example, cholera toxin and Shiga toxin undergo retro-translocation to the ER, where they fold appropriately before arriving at their final destinations in the IECs. In the ER lumen, the A subunits of cholera toxin (CTA) and Shiga toxin (Stx1A) activate NF-*κ*B through direct binding to IRE1*α* and subsequent activation of RIDD [126]. The UPR activation by pathogens and their molecules also involves the pattern recognition receptors including Toll-like receptors (TLRs) and nucleotide-binding oligomerization domain- (NOD-) like receptors (NLRs). In murine and human macrophages, ligands of TLR2/4 specifically activate the IRE1*α*-XBP1 pathway that requires recruitment of TNF receptor-associated factor 6 (TRAF6) to the TLR and subsequent ROS production by NADPH oxidase NOX2. The spliced XBP1 then induces the expression of *Tnfα* and *Il6* by binding to their promoter regions [127]. IRE1*α* is known to activate the JNK-dependent inflammatory signaling through the recruitment of TRAF2 [128]. A recent study showed that the interaction between TRAF2 and NOD1/NOD2 is indispensable in this process [129]. This is of particular interest because some genetic variants of both *NOD2* and *XBP1* have been implicated in Crohn's disease [10]. So far, most studies have focused on the interactions between pathogenic bacteria and the ER. There is lack of data on how commensal bacteria affect the ER function and homeostasis.

Viral invasion and replication can active the UPR in host cells. At least 36 eukaryotic viruses have been shown to trigger ER stress and induce downstream pathways including inflammatory and immune responses [130]. However, little is known about how gut virome affects the ER homeostasis in any cell type in the gastrointestinal tract. For the last decade, the findings that helminth infection may improve IBD have drawn our attention to the role of parasites in intestinal health [131, 132]. A recent study showed that infection of *Schistosoma japonicum* mitigated DSS-induced colitis in mice. Alleviated ER stress in the colonic tissues was shown to be partly responsible for the improved inflammation and mucosal cell apoptosis in infected mice, although the underlying mechanisms are unclear [133].

### 3.2. Oxidative Stress and Gut Microbiota

Oxidative stress in the gastrointestinal tract plays a multifactorial role in the pathogenesis of IBD. Previous studies have associated elevated ROS with dysbiosis in the gut. Given the radial oxygen gradient in the intestinal tract, bacteria residing on the colonic mucosa have higher oxygen tolerance and catalase expression relative to their luminal or stool-associated counterparts [134]. As inflammation usually drives an oxidative state, it might favor the outgrowth of aerotolerant phyla such as Actinobacteria and Proteobacteria in the gut, one example is the murine pathogen *Citrobacter rodentium* [135]. Furthermore, intestinal inflammation has been shown to upregulate the production of small molecules that function as terminal electron acceptors for facultative anaerobes such as Enterobacteriaceae [136, 137], thus contributing to dysbiosis in the intestine.

During intestinal inflammation, the gut microbiota can contribute to ROS production directly and/or indirectly through the mucosal cells [43] (Figure 1). One example is *H. pylori* that generates ROS both by itself and by inducing neutrophils to produce ROS [138]. Some bacteria in the gut can enhance the production of nitric oxide and nitrous oxide by activating macrophages in the settings of inflammation-induced DNA damage [139, 140]. Importantly, the host DNA repair mechanisms can be altered by bacterial modulation. Some strains of enteropathogenic *E. coli* have been shown to interfere with the DNA mismatch repair by secreted toxins, leading to apoptotic cell death and carcinogenesis [141, 142].

### 3.3. Hypoxic Stress and Gut Microbiota

The gut microbiota influence intestinal health in a number of ways, one of which is the production of short-chain fatty acids (SCFA) including butyrate, propionate, and acetate [143]. In addition to being absorbed into the circulation, SCFAs serve as energy sources of colonocytes and are important for intestinal barrier function. Butyrate augments regulatory T cell function by activating G-protein coupled receptors and leads to epigenetic modification through the inhibition of histone deacetylases [97]. In addition, metabolism of butyrate has recently been shown to boost the oxygen consumption and to stabilize HIFs in murine IECs. Consistently, the level of hypoxia is lower with decreased expression of HIFs in germ-free mice [143]. Depletion of butyrate-producing Clostridia using antibiotics facilitated the expansion of *Salmonella* in the more aerobic (or less hypoxic) mucosal microenvironment [144]. These data indicate that the gut microbiota composition dramatically impacts intestinal oxygenation versus hypoxia status, which orchestrates the composition of aerobes versus anaerobes and gut dysbiosis. A recent study linked the peroxisome proliferator-activated receptor-*γ* (PPAR*γ*) to the bioavailability of oxygen in the microbial composition and intestinal homeostasis. Butyrate in the colon activates the nuclear receptor PPAR*γ*, which drives the *β*-oxidation of butyrate in the colonocytes. This process is highly oxygen-consuming and thereby generates a hypoxic environment that limits the expansion of facultative anaerobic bacteria including the Enterobacteriaceae [145]. The ileal mucosa of patients with CD is predominantly colonized by adherent-invasive *Escherichia coli* (AIEC) [146], which requires the interaction between type 1 pili on AIEC and CEACAM6 receptors on the IECs [147]. Another study revealed potentially methylated dinucleotide CpGs within the hypoxia-responsive elements (HREs) of CEACAM6 promoter and the inverse correlation between the methylation and CEACAM6 expression in the IECs [148]. This data highlight a role of HRE methylation and HIF-1 in the regulation of AIEC colonization and ileal mucosal inflammation. Furthermore, hypoxia is a known regulator of autophagy and NLR signaling, two key innate immune mechanisms associated with IBD. A recent study showed that hypoxia suppresses NF-*κ*B and NLRP3 signaling via hypoxia-induced autophagy in IECs, thereby relieving colitis in mice [149]. This data suggests that hypoxia is an important regulator of the microbial-epithelial interaction and innate immunity in the gut (Figure 1).

Importantly, the luminal oxygen level diminishes significantly from the small intestine to large intestine, together with other factors such as antimicrobial peptides and pH, creating distinct habitats for commensal bacteria. Fast-growing facultative anaerobes that effectively utilize simple carbohydrates while tolerating oxidative stress, bile acids, and antimicrobial peptides dominate in the small intestine [150]. For example, certain *Clostridium* spp. and some members of the phylum Proteobacteria are enriched in ileostomy samples from humans [151]. In contrast, the cecum and colon cultivate a more diverse community of bacteria. Fermentative polysaccharide-degrading anaerobes, notably the Bacteroidaceae and Clostridia, flourish in the large bowel thanks to its more hypoxic environment, slower transit time, a lack of simple carbon sources, and lower levels of antimicrobials [152]. The longitudinal gradient of oxygenation plays an important role in the intestinal homeostasis, either dependent or independent of the microbiota. It remains unclear whether the hypoxia gradient along the GI tract contributes to the longitudinal distribution of disease in UC versus CD.

## 4. Discussions

The complex pathophysiology of IBD is linked to the tremendous complexity of interactions between the host cells and microorganisms in the gastrointestinal tract. Gut microflora is a key component of the mammalian digestive system and has been shown to play a critical role in intestinal health in humans. Although the causative relationship between dysbiosis and the pathogenesis of IBD has not yet been established, data from both animal and human studies suggest that the altered composition and metabolic profiles are not merely a byproduct of intestinal inflammation. It is evident that gut microbiome can influence intestinal homeostasis by interacting with multiple types of cells in the gut through numerous signaling pathways. During evolution, cellular stress responses have developed as important players in the cross-talks with gut microorganisms, an essential component of environmental stress one faces on a daily basis. Both *in vitro* and *in vivo* studies have shown how gut microorganisms and their molecules activate cellular stress pathways, which can produce distinct effects on cell fate and tissue homeostasis. Recent studies found that the ER stress/UPR, antioxidative stress response, and hypoxic stress response may contribute to IBD via multiple mechanisms such as changes in cellular metabolism, altered differentiation and survival of cells, epithelial barrier impairment, proinflammatory response, and fibrosis and wound healing. The picture is further complicated by the fact that ER stress, oxidative stress, and hypoxia interact extensively during intestinal inflammation [10, 153, 154]. Future studies should focus on the network of cellular stress responses in the interaction with gut microbiome using tools such as systems biology.

There is evidence from animal studies that deletion of a single gene associated with the stress signaling (e.g., *Xbp1*) or transfer of colitogenic bacteria to wild-type mice is sufficient to cause inflammation [11, 155, 156]. However, such examples are sparse in humans and the vast majority of IBD cases are probably multifactorial involving both host genetics and environmental factors. It is possible that an altered stress response from either genetic or epigenetic defects can initiate clinical disease of IBD, when the individuals encounter certain microorganisms/molecules in the gut that target these pathways and/or their compensatory pathways directly or indirectly. One example of this susceptibility gene-microbial interaction in mice is that a combination of impaired autophagy (from a mutated *Atg16L1*) and colonization of murine norovirus results in Paneth cell dysfunction, resembling the abnormalities seen in some patients with Crohn's ileitis [157]. Microbiome GWAS (mGWAS) has shed light on how host genetics may interact with gut microbiota in IBD pathogenesis by combining human genome sequencing and 16s rRNA or metagenome sequencing of microbiome [158]. Using these tools, the human lactase gene *LCT* was associated with the abundance of *Bifidobacterium* in a small mGWAS and validated in subsequent cohorts [159]. Future mGWAS with larger sample sizes are likely to achieve higher statistical power [154]. Longitudinal studies that start tracking genetically susceptible individuals before the onset of clinical disease may shed light on the causality between certain microorganism populations and IBD. Following this strategy, a recently published study showed an alternation of intestinal virome that occurred before the development of type-1 diabetes-associated serum auto-antibodies in genetically susceptible children [160].

Bacteria are by far the most studied microorganisms in the gut. Some gastrointestinal pathogens are known to disturb ER function and modulate the UPR either by physically invading the ER lumen or via their secreted molecules. However, how specific gut commensal populations influence host's stress signaling remains elusive. It is likely that the interactions between the host and the commensal microorganisms follow rules different from those between the host and pathogens. Given the findings that certain bacterial species can trigger intestinal inflammation upon transfer to wild-type mice, it would be worthwhile to explore how these microorganisms affect cellular stress responses in the gastrointestinal tract. In addition, some bacterial metabolites can affect mucosal homeostasis via interactions with IECs and immune cells. Future studies should explore how these molecules cross-talk with the stress signaling pathways in these cells. While many mutations in the stress pathways alone do not cause clinical disease in mice, these models provide unique opportunities to study the interactions between susceptibility genes and specific populations of the microbiome in intestinal inflammation. While many human viruses have been shown to compromise ER function during their invasion and/or replication, the relevance of gut virome to ER stress or other stress pathways is unknown. Similarly, the potential interactions between the stress pathways and fungal species in the gut remain to be determined. The exploration into these puzzles will not only further our understanding of IBD but also spur the efforts in developing novel therapies for this disease.
